# How Adequate Are the Guidelines for Dietary and Workplace Exposure to Cadmium?

**DOI:** 10.3390/toxics14050408

**Published:** 2026-05-08

**Authors:** Soisungwan Satarug

**Affiliations:** Centre for Kidney Disease Research, Translational Research Institute, The University of Queensland, Woolloongabba, Brisbane, QLD 4102, Australia; sj.satarug@yahoo.com.au

**Keywords:** cadmium, bone and kidney targets, exposure limits, health risk, mitigation of toxicity, reference dose, tolerable intake level, toxicological reference value

## Abstract

Cadmium (Cd) is a heavy metal pollutant to which most people are exposed daily through their diet because of its presence in nearly all food types, including potatoes, vegetables, cereals, grains, legumes, shellfish, and organ meat. Cd has no physiological role or nutritional value in the body and causes toxicity to multiple tissues and organs via oxidative stress and chronic inflammation; as such, at high prevalence, it is frequently associated with diseases, notably cancer, heart disease, diabetes, osteoporosis, and chronic kidney disease. Using kidneys and bones as critical toxicity targets, current dietary Cd exposure guidelines vary from 0.21 to 0.83 μg/kg b.w./d. There is a widespread concern about these guidelines because they were based on the excretion of β_2_-microglobulin (β_2_M) at a rate of 300 µg/g of creatinine as an endpoint. Concerningly, rice is a staple food for over 50% of the world’s population; however, the permissible Cd level in this commodity has not been adequately addressed. This narrative review focuses on critiquing existing food standards and exposure guidelines for Cd. It discusses the threshold-based risk assessment that was used to define the no-observed-adverse-effect level (NOAEL) for Cd, when β_2_M excretion was used with Cd excretion at a rate of 5.24 µg/g of creatinine being a threshold. The estimated glomerular filtration rate (eGFR) is recommended as an appropriate kidney disease endpoint. The current view around how Cd uses various transport proteins to enter and induce toxicity to its target cells are summarized. The strategies to minimize Cd accumulation and mitigate its nephrotoxicity are highlighted.

## 1. Introduction

Exposure to pollution from environmental cadmium (Cd) has been linked to worldwide rising prevalences of ill-health conditions, malignant and non-malignant diseases [1,2,3]. Hypertension [4] and iron deficiency anemia [5] are poor health conditions, while osteoporosis [6,7,8], type 2 diabetes [9,10] and chronic kidney disease (CKD) [11,12] are consistently found to be associated with environmental Cd exposure.

CKD is a highly prevalent disease, affecting 8–13% of adult population worldwide, reaching epidemic proportions in many parts of the world [1,2]. It is diagnosed when the estimated glomerular filtration rate (eGFR) falls to one-third of the normal range and/or the presence of albuminuria for at least 3 months [1,2]. A fall of eGFR at a high rate (≥3 mL/min/1.73 m^2^ per year) has been causally linked to environmental Cd exposure in a prospective cohort study from Switzerland (*n* = 4704, mean age 51.9 years, a mean follow-up 12.5 years) [13]. Using the Bradford–Hill Criteria, Hsu et al. presented evidence that strengthens causal relationships between Cd exposure and kidney and bone diseases in the general population [3].

Cd is a cumulative toxicant because its excretion rate is minuscule, resulting in a long half-life, varying from 7.4 to 30 years [14,15,16]. It preferentially accumulates within kidney tubular cells and is released into the urine as tubular cells die from any cause [17]. For this reason, Cd excretion is a reliable quantitative measure of cumulative exposure or the body burden of the metal [17,18]. Cd can cause damage to target cells via multiple mechanisms, such as oxidative stress and depletion of antioxidant defenses [1,2]. It can also induce functional iron (Fe) and zinc (Zn) deficiencies [5,13,19,20].

Concerningly, however, the health risk of Cd exposure has long been underappreciated as a result of using an inappropriate toxic endpoint in health risk estimation. Most frequently, an elevation in β_2_-microglobulin (β_2_M) excretion was used for such purposes [21,22]. As another concern, at-risk subpopulations, namely, children, women of childbearing age, and those with marginal Zn intake levels and low body Fe stores, have not been considered in computing the Cd health risk. Because of enhanced intestinal absorption of metals and immaturity of the blood–brain barrier, neonates, infants, and young children are more susceptible than adults to adverse effects of food-borne toxicants [23,24]. Moreover, relative to their body size, food intake is typically larger than that of adults; consequently, they are exposed to a higher dose of ingested toxicants [25,26].

**Figure 1:**
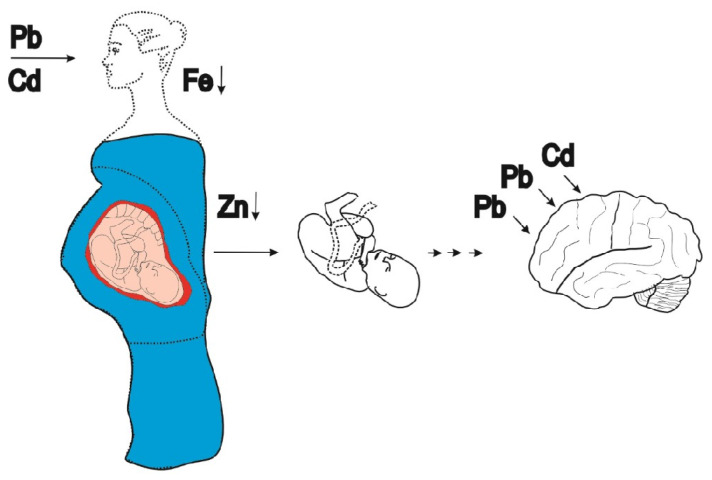
Sup-population groups with increased susceptibility to cadmium toxicity. The passage of Cd from mother to fetus is negligible because of the placental barrier [27]. Pb is readily transferred to fetus, especially when body content of Fe and Zn are low (↓). No comparable barrier exists in the mammary glands; consequently, Cd is secreted in breast milk [31,32,33].

Through the interference with mother-to-fetus Zn transport, maternal Cd exposure has been associated with infant low birth weight and neurodevelopment deficits, even though the passage of Cd from mother to fetus is minimal [27,28,29,30]. The ability of Cd to inhibit calcium (Ca) secretion by mammary glands has also been observed [31], along with the secretion of Cd into breast milk [31,32,33], raising concern about the potential for early-life Cd exposure, which can impact disease risk in adulthood [34,35,36] (Figure 1).

At present, around 15% of the world’s cultivation soils are contaminated with toxic metals, especially Cd, which is particularly prevalent in South and East Asia and parts of the Middle East and Africa [37]. Hence, there is a widespread concern that dietary Cd exposure in the population will gradually increase because Cd, being non-biodegradable, can persist indefinitely in soils, facilitating entry into the human food chain and its ubiquitous presence in our diet, especially staple foods. As Cd soil-to-plant transference continues, as does human exposure to the metal in the diet, the prevalence of Cd-related diseases will eventually reach an epidemic proportion.

This narrative review provides information relevant to public health policy regarding “safe” Cd exposure levels that are much lower than officially estimated. It calls for a paradigm shift in the criteria on which the toxicity of Cd is based. It highlights the inadequacy of existing food standards and exposure guidelines and underscores a conceptual flaw in using β_2_M excretion in toxicological risk assessment practice.

It underscores the use of advanced benchmark dose (BMD) modeling [38,39,40], especially in determining the BMD limit (BMDL) values, using eGFR decline as an endpoint [3,41,42]. Cd-induced eGFR loss reliably signifies CKD and its progression toward kidney failure. The advanced BMD modeling overcomes some shortcomings of the conventional toxicological risk assessment, involving the identification of a point of departure (POD) from a dose–response curve [43,44].

Additionally, mechanism-based mitigation for Cd toxicity to its target cells, involving exogenous heme oxygenase-1 (HO-1) inducers, is explored as a potential strategy to delay CKD progression.

## 2. Cadmium Exposure Limits and Toxicity Thresholds

**Figure 2:**
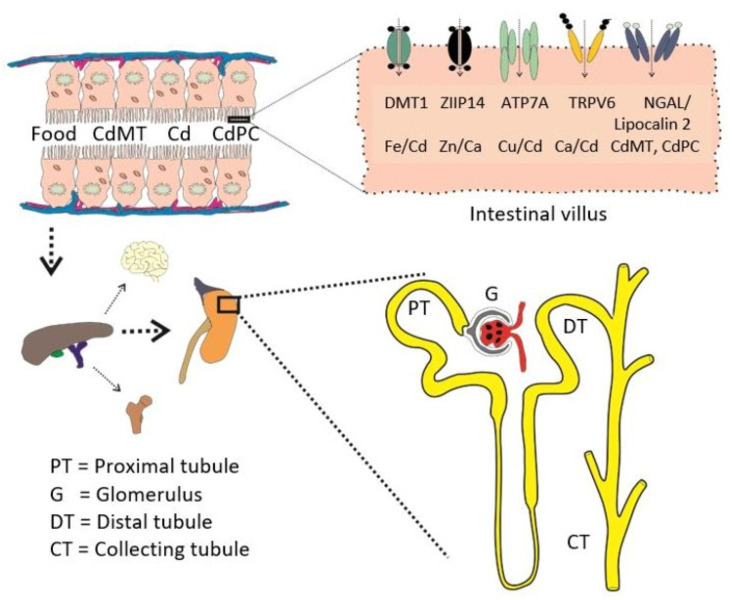
The pathways for cadmium from foods to bone and kidney targets. Multiple metal transport proteins are involved in Cd absorption, including those for Fe (DMT1) [45], Zn (ZIP14) [46,47], copper (ATP7A) [48] and calcium (TRPV6) [49,50]. Thus, Cd is absorbed at a rate higher than that of Fe, Zn, and Ca, which can be enhanced further in those with low body iron stores, more prevalent in women than men, as well as in children than adults [1,2]. Also, the SLC22A17 or human neutrophil gelatinase-associated lipocalin (hNGAL)/lipocalin-2 receptor facilitates the assimilation of Cd complexed with metallothionein (MT) and the plant metal-binding ligand phytochelatin (PC), denoted as CdMT and CdPC, respectively [51,52].

In this section, environmental standards for Cd are highlighted along with a summary of toxicological risk assessment methodology that was used to define “safe” exposure levels. Presumably, intake of Cd from drinking water was insignificant, given that Cd levels in most portable water are below the Australian/New Zealand standard of 2 µg/L and the US EPA and WHO of 5 µg/L [43]. Therefore, exposure limits were determined based on its existence in the human diet, a principal source of Cd for most people. For workers, exposure limits were determined based on inhaled Cd that can enter the circulation from the lungs [43]. Figure 2 depicts pathways for Cd in food to its targets, e.g., bones and the proximal tubular cells of the kidneys.

### 2.1. Food and Environmental Safety Standards

The Codex Alimentarius set a permissible maximum level (ML) of Cd in rice at 0.4 mg/kg dry grain weight [53]. The European Food Safety Authority (EFSA) set the ML for Cd in this commodity at 0.2 mg/kg [54]. Rice contributed up to 90% of Cd exposure in an area of Vietnam with Cd contamination [55]. In comparison, rice and its products contributed 40–50% to dietary Cd exposure among women living in Cd-contaminated areas of Japan [56]. In Portugal, bread was identified as the main dietary source of Cd [57]. Potatoes contributed most to Cd intake by 1- and 2-year-old Dutch children [58]. Data from the Chinese National Diet and Nutrition Survey and the National Food Contamination Monitoring Program supported the ML for rice below 0.2 mg/kg [59]. Severe damage to kidneys and bones, such as that in Itai-Itai disease patients, may have occurred following ingestion of rice with Cd content of 0.27 mg/kg [60].

### 2.2. Toxicity Threshold Definition

A toxicity threshold is referred to as the highest dose that does not produce an adverse effect in the organ most sensitive to a health-hazardous substance of concern [43,44]. Thus, for a food contaminant like Cd that impacts multiple organs, a health-protective exposure guideline must be based on the most sensitive endpoint.

### 2.3. Official Dietary Exposure Guidelines

“Safe” dietary Cd exposure levels were derived from various international agencies, including the Food and Agriculture Organization and World Health Organization (FAO/WHO) Joint Expert Committee on Food Additives and Contaminants (JECFA) [61,62], EFSA [63], the US Food and Drug Administration (US FDA) [64] and the Agency for Toxic Substances and Disease Registry (ATSDR) [43].

Notably, however, different terms were used to describe the acceptable dietary exposure levels of Cd; nevertheless, most countries have adopted JECFA’s tolerable daily intake (TDI) and a threshold at urinary Cd excretion rate of 5.24 μg/g creatinine (Table 1).

#### 2.3.1. JECFA/TDI

JECFA described the exposure guideline for any food contaminant as a provisional tolerable weekly intake (PTWI), meaning an estimate of the amount of a chemical with no intended function that can be ingested weekly over a lifetime without appreciable health risk [61,62]. The JECFA’s original PTWI for Cd was 400–500 µg/person/wk, revised to 7 µg/kg b.w./wk [61] before being 25 µg/kg b.w./m (TMI), equivalent to 0.83 μg/kg b.w./d (TDI) [62]. The PTWI, TMI, and TDI values for Cd were based on a permissible lifetime intake of 2 g of Cd, and β_2_M excretion rate at 300 µg/g of creatinine.

The Food Safety Commission of Japan has adopted the PTWI for Cd at 7 µg/kg b.w./wk (doi: 10.14252/foodsafetyfscj.D-24-00011) for the reason that the estimated dietary Cd intake in Japan in 2022 was 2.03 μg/kg b.w./wk, 29% of the PTWI figure.

The JECFA’s assumption of a “safe” lifetime intake level of Cd of 2 g was challenged by a prospective cohort study showing that a lifetime Cd intake of 1 g may have increased the mortality from kidney failure by 49% in women residing in a Cd-contaminated area of Japan [65]. The mortality risk was adjusted for potential confounders.

The proportion of the Portuguese population 18–74 years of age with Cd exposure levels higher than JECFA’s TDI was 5.4%, even though mean dietary Cd intake was low (0.19 µg/kg b.w./d) [56]. In comparison, the mean dietary Cd intake in the Chinese adult population was 34.3 µg/day (range: 22.6–54 μg/d), of which 15.4% had dietary Cd exposure levels exceeding JECFA’s TDI [66]. Fungi and algae had the highest Cd contents, followed by aquatic foods, nuts, cereals, beans, vegetables, meats, eggs, milk, and fruits.

Like Cd, lead (Pb) is another ubiquitous food contaminant for which the PTWI was derived as 25 μg/kg b.w./wk [61]. However, this guideline was withdrawn because of the absence of a threshold for Pb-induced neurotoxicity, meaning that no intake amount of Pb carries a negligible health risk [62].

#### 2.3.2. EFSA/RfD

EFSA considered the kidneys to be the critical Cd toxicity target and used β_2_M excretion at a rate of 300 µg/g of creatinine as an endpoint. However, EFSA designated Cd excretion at a rate of 1 μg/g creatinine as a threshold level after an uncertainty factor (a safety margin) was included in a model to compensate for the variation in dietary Cd exposure levels among people [63]. EFSA designated dietary exposure to Cd at 0.36 μg/kg bw/d for 50 years as an acceptable dietary Cd exposure level and described it as a reference dose (RfD) [63].

#### 2.3.3. US FDA/TRV

Dietary Cd exposure limits derived by the US FDA ranged between 0.21 and 0.36 μg/kg bw/d, assuming Cd excretion at a rate of 0.5 μg/g creatinine as a threshold level for both bone and kidney targets [64]. These US FDA’s acceptable exposure levels for Cd were called toxicological reference values (TRV), obtained through reverse dosimetry methodology with the physiologically based pharmacokinetics model [64].

Among US children aged 1–6 years, the food groups contributing most to Cd exposure were grains/baking, dairy and fruit and grains/baking and vegetables [67]. Respective mean value and 90th-percentile level for dietary Cd exposures were 0.43 and 0.71 μg/kg b.w./d, both of which were higher than the US FDA’s TRV but were within the JECFA’s TDI.

Because a threshold could not be established for the neurotoxicity of Pb, the US FDA has proposed a dietary Pb intake level of 12.5 μg/d as an interim dietary exposure guideline for adults [68]. This Pb exposure rate corresponds to a blood Pb concentration of 0.5 μg/dL, the internal dose that has not been associated with any adverse effect in adults [69].

#### 2.3.4. ATSDR/MRL

ATSDR derived Cd exposure guidelines, known as minimal risk levels (MRL), using data from experimental animal dosing regimens [43]. With the bone target, an MRL of 0.5 µg/kg b.w./d was obtained for oral Cd exposure in an intermediate exposure duration (15–365 d). Data were from Wistar rats exposed to Cd as CdCl_2_ in drinking water at 0, 1, 5, or 50 mg/L for 6, 9, or 12 m [70,71,72].

With the lung target, an MRL value of Cd as Cd oxide (CdO) of 0.03 μg/m^3^ was obtained for acute inhalational exposure for 1−14 d [43]. Data were from Fisher F344 rats exposed to CdO at 0, 0.1, 0.3, 1, 3, or 10 mg CdO/m^3^ for 6.2 h/, 5 d/wk, for 2 wks [73].

### 2.4. Other Exposure Limits and Thresholds Derived for Oral Cd

Reported Cd exposure limits and thresholds are abundant, and they are highly variable, depending on methodology, demographic characteristics of study populations, toxicity targets and endpoint measures. A few studies are summarized below.

Sweden: Using data from 794 Swedish women, aged 53–64 years, Suwazona et al. identified a urinary Cd excretion rate of 1 µg/g of creatinine as a threshold for the osteoporosis endpoint [74]. This Cd excretion rate is the same as EFSA’s threshold for kidney effects (β_2_M endpoint) [63].

France: Leconte et al. derived oral Cd of 0.35 μg/kg b.w./d as an exposure limit figure for the French population, with Cd excretion at a rate of 0.5 μg/g creatinine being a threshold for decreased bone mineral density [75].

China: Qing et al. derived dietary Cd exposure limits, using Chinese population data with an average Cd exposure of 30.6 μg/d. For the bone target, the TDI value was 0.64 μg/kg b.w./d, and the Cd excretion rate of 1.71 μg/g creatinine was a threshold [76]. For the kidney target, the TDI value was 0.28 μg/kg b.w./d (16.8 µg/d for a 60 kg person), and Cd excretion rates of 3.07 and 2.93 μg/g creatinine were the threshold levels for the β_2_M and N-acetyl-β-D-glucosaminidase (NAG) endpoints, respectively [77].

Experimentation: Human TDI value for oral Cd exposure was 0.2 μg/kg b.w./d, using data from inbred pigs, exposed to Cd at 0, 0.5, 2, 8, or 32 mg Cd/kg of feed for 100 d [78]. This TDI was obtained after inclusion of an uncertainty factor of 100 to extrapolate data from pigs to humans. Unexpectedly, abnormal β_2_M excretion occurred at the highest Cd feeding dose, while abnormal excretion of retinal binding protein (RBP) was observed at the lowest Cd-dose level. The oral Cd dose levels resulting in abnormal excretion rates of RBP, NAG, Cd complexed with metallothionein (CdMT), and β_2_M were 0.67, 0.88, 1.00, and 3.08 mg/kg feed, respectively. Thus, β_2_M excretion was least sensitive to Cd, compared to RBP and NAG. These data cast considerable doubts on the use of β_2_M excretion as a basis to derive Cd exposure limits.

### 2.5. Cadmium Inhalational Exposure Limits for Workers

Similarly, the kidney target and β_2_M endpoint are used in the assessment of health risk from workplace exposure, which mostly involves an inhalational route [79]. As revealed in studies from Japan [80,81] and Korea [82], workers’ exposure limits at blood Cd concentration of 5 µg/L and Cd excretion at a rate of 5 μg/g creatinine did not provide sufficient protection against Cd toxicity to the kidneys. The authors concluded that current workers’ Cd exposure limits should be lowered, while monitoring and management of exposures among workers remain necessary [80,81].

Nogawa et al. [80] used data from 326 male and 114 female Japanese workers, and they estimated the BMDL value for a 40-year cumulative inhalational exposure to Cd to be 17.7 µg/m^3^. In addition, they found the warning blood Cd concentration to be between 1.8 and 2.0 µg/L, less than half of the current exposure limit of 5 µg/L [80]. Hoshino et al. analyzed data from 238 workers at two nickel-Cd battery plants in Japan; they observed that the risk of abnormal β_2_M excretion was increased 17% even though the geometric mean blood Cd level among workers was 1.97 μg/L [81]. This blood Cd level associated with abnormal β_2_M excretion in workers was similar to that reported by Nogawa et al. [80].

Choi et al. analyzed data from Korean workers of a small-scale silver soldering company who were exposed to air Cd concentrations of 6–15 µg/m^3^ [82]. They observed alarmingly high Cd excretion rates [mean (range) of 22.15 (3.23–62.97) μg/g creatinine] together with elevated urinary concentrations of β_2_M and total protein [82].

In a case report, a male jewelry worker developed hypophosphatemic osteomalacia following exposure to a high dose of Cd in fumes [83,84]. His blood Cd level was 6 times higher than the permissible workplace exposure level at 5 µg/L, while his 24 h urinary Cd excretion was 51 µg [83]. He also had hypochromic microcytic anemia, most likely from Cd-induced functional iron deficiency [5,20,85], plus an elevation in circulating levels of the bone-derived hormone, fibroblast growth factor 23 (FGF23), which suppresses erythropoietin synthesis in the kidneys, while reducing kidney tubular reabsorption of phosphate [86,87].

### 2.6. Specific Recommendations for Policy Makers

Using Cd concentrations in foods, estimated Cd exposure from the diet among average and high consumers was 30 and 93.5 µg/d, respectively [88] (Table 2).

Foods that are frequently consumed in large quantities, like cereals, pulses, legumes, rice, and wheat, contribute the most to Cd exposure [88]. Therefore, it is recommended that the permissible Cd levels in food crops and feeds should be set at the lowest achievable levels, e.g., 0.05–0.10 mg/kg for rice, potatoes, and wheat.

Exposure guidelines should be developed for children, pregnant women, and individuals with iron/zinc deficiency who are more sensitive to Cd. The cumulative feature of Cd means that a critical exposure level (threshold) may not exist. This is evident from a meta-analysis, where a nearly two-fold increase in risk of osteoporosis was found in both low- and high-Cd exposure groups [89].

Excretion of Cd signifies nephrotoxicity in progress, not predicting future health risk [17,78]. This is because excreted CdMT and NAG both emanate from kidney tubular cells. Urinary CdMT has long been misinterpreted as the filtered CdMT, which was not reabsorbed by kidney tubular cells. The misconception and its ramifications are discussed by Thévenod and Lee, along with the cutting-edge knowledge on Cd nephrotoxicity [90].

For an accurate health risk computation, the interindividual differences in functioning nephrons and muscle mass should be eliminated by normalizing the excretion of Cd and nephrotoxicity indicators, namely, NAG and RBP, to creatinine clearance (C_cr_) instead of creatinine excretion (E_cr_) [42].

## 3. Cd Excretion Threshold Levels Based on Disease Endpoints

Data from studies on Cd exposure and adverse health effects in the general populations and metal workers (Section 2) have repeatedly indicated the inadequacy of existing food standards and official exposure guidelines to protect the population’s health.

In this section, the derivation of the critical exposure level/thresholds, known as the benchmark dose (BMD) limit (BMDL), is discussed, focusing on kidney disease manifestation of Cd exposure in comparison to nephrotoxicity indicators, like β_2_M, RBP and NAG [91].

### 3.1. Excretion of Low-Molecular-Weight Proteins: Tubular Proteinuria

Excretion of β_2_M and other low-molecular-weight proteins, namely, α1-microglobulin (α1M), and RBP, has been used in assessing Cd effects [1,2]. These proteins, with a molecular weight <20 kDa, readily pass through the glomerular membrane into the tubular lumen, are reabsorbed, and are degraded within proximal tubular cells of the kidney [91].

**Figure 3:**
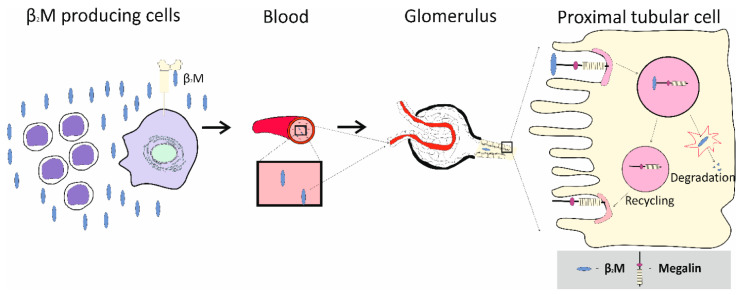
Biosynthesis and the catabolism of β_2_-microglobulin. The protein β_2_M is released into bloodstream from the surface of most nucleated cells, including white blood cells [12,92]. With the low molecular weight of 11.8 kDa, β_2_M is filtered by the glomeruli, retrieved by the proximal tubular cells (PTCs), and degraded in lysosome. Increased β_2_M excretion can be due to enhanced synthesis, impaired reabsorption/catabolism, or nephron loss. This figure is from Phelps et al. https://dx.doi.org/10.20517/jeea.2025.09 (accessed on 17 March 2026) [93].

In theory, the excretion rate of any of these proteins is a function of its synthesis, glomerular filtration rate, kidney tubular reabsorption, and degradation. Thus, a rise in its production for any reason, a fall in eGFR due to nephron loss, defective tubular reabsorption, or degradation, can all lead to an increase in its excretion (Figure 3).

A close examination of the parameters influencing β_2_M homeostasis in Thai subjects, exposed to low levels of Cd, has revealed that β_2_M excretion could not be used as a measure of tubular dysfunction [93]. The variation among people in the influx of β_2_M from cells into plasma is so large that β_2_M excretion is unrelated to its reabsorption and degradation by kidney tubules. There is no basis to estimate permissible dietary Cd exposure levels using β_2_M excretion at a rate as high as 300 µg/g of creatinine.

### 3.2. From NOAEL to BMDL

The practice of toxicological risk assessment involves determining from a dose–response curve a point of departure (POD), which represents the dose at which a specific adverse effect is *first* observed, or at which a response *deviates* from a baseline or control [43,44]. POD serves as a starting point to evaluate the potential health impact of exposure to a hazardous substance.

Typically, the POD figure is determined from a dose–response curve, constructed from experimental dosing, which often involves daily administration of 4–5 different doses for 90 days or longer [94,95,96]. From a dose–response curve, a POD may be established from the lower bound “no-observed-adverse-effect level” (NOAEL) and the upper bound “lowest-observed-adverse-effect level” (LOAEL).

The NOAEL value is referred to as the highest dose tested that produces an insignificant effect compared to controls. To convert NOAEL to the BMD lower limit (BMDL), the NOAEL is divided by an uncertainty factor that accounts for species differences and human variability [43,44]. The incorporation of an uncertainty factor to extrapolate data from pig dosing experimentation obtained a Cd exposure limit 4 times lower than the JECFA’s TDI [78] (Section 2.4).

A clear dose–response relationship is a prerequisite to estimate the NOAEL (BMDL) figure for exposure to any health-hazardous substance. However, reliance on a single mathematical dose–response model (equation), such as the Hill model, can lead to erroneous estimates of the NOAEL (BMDL) values of Cd excretion, as is the use of cut-off values to define abnormal excretion rates of nephrotoxicity biomarkers.

### 3.3. Multiple Mathematical Dose–Response Models: The Akaike Information Criterion (AIC)

The advanced BMD method involves fitting an entire exposure–effect dataset to multiple mathematical dose–response models, in which a specific effect size, termed benchmark response (BMR), is pre-defined [43,44,96]. In PROAST BMD software, version 71.1, four mathematical dose–response (MDR) models and seven MDR models are used for fitting continuous dose-effect data and dichotomous data, respectively [38,39,40,96].

The Akaike information criterion (AIC) evaluates how well each model fits the data [96]. The AIC accounted for both goodness of fit and model simplicity, thereby balancing the risk of overfitting and underfitting of the data. The model weight is relative to the amount of information lost by a given model: the less loss, the higher the weight and the higher quality of that model. The shape and steepness of the slope provide additional insight into exposure-effect pairs [38,39,40].

The BMDL value of any exposure indicator is defined as the lower 95% confidence bound of the BMD, computed at a 5% BMR. The BMDL value derived in this manner has replaced NOAEL, which can serve as a representative of a critical exposure level [44,96].

Bootstrapping with 200 repeats or more is used to determine BMD and 95% confidence intervals [96]. The difference between the lower bound (BMDL) and upper bound (BMDU) of the 95% confidence interval (CI) of the BMD reflects the statistical uncertainties in the BMD estimates. A narrow difference indicates a high degree of certainty of the estimated BMD figures. Conversely, a wide difference, e.g., a BMDU/BMDL ratio ≥ 100, indicates unreliable BMD estimates [44].

#### 3.3.1. BMDL Values of Cd Excretion Derived from the β_2_M vs. eGFR Endpoints

A population of people, exposed to a wide range of Cd doses, is required to derive BMDL values of Cd excretion rates for various nephrotoxicity endpoints. Accordingly, the Mae Sot District in Western Thailand, where Cd pollution is endemic [97], has provided a well-circumscribed population of people with dietary exposure levels that would enable assessment of the health impact of Cd exposure.

The Cd concentration of the paddy soil samples from the area was higher than the Thailand standard of 0.15 mg/kg, and the rice samples collected from household storage contained four times the standard Cd level of 0.1 mg/kg [98]. An inverse association was observed between excretion of β_2_M and eGFR only in those with eGFR values within the CKD signified range, and risk of CKD rose 4.7-fold as β_2_M excretion rose from 100 to 300 μg/g cr [99]. Thus, an elevation of β_2_M excretion could be indicative of nephron loss, which caused a fall in eGFR [99]. In a dose–response analysis, CKD risk rose 4.7-fold, 6.2-fold, and 10.5-fold at β_2_M excretion rates of 100–299, 300–999, and ≥1000 μg/g cr, respectively [100].

To reassess the Cd excretion threshold based on β_2_M excretion, in comparison with the eGFR reduction, Satarug et al. employed data from 799 Thai nationals, 18–87 years of age, selected from a large cohort of the residents of low-exposure areas and moderate-to-high exposure locations of the Mae Sot District [101].

**Figure 4:**
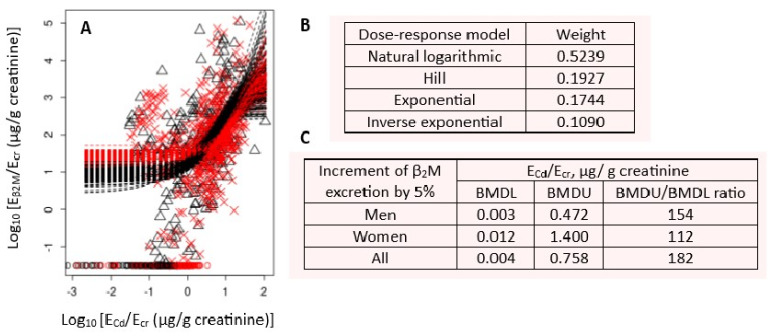
Cd excretion rate associated with a 5% increase in β_2_M excretion. Bootstrap dose–response model averaging with 200 repeats for E_β2M_/E_cr_ − E_Cd_/E_cr_ dataset (**A**), dose–response models and model weights (**B**), and BMDL/BMDU values for Cd excretion rates (**C**). × and △ represent male and female participants, respectively. Data were from 799 subjects (300 men and 499 women), aged 19–87 years (mean of 49.2 years) [42].

**Figure 5:**
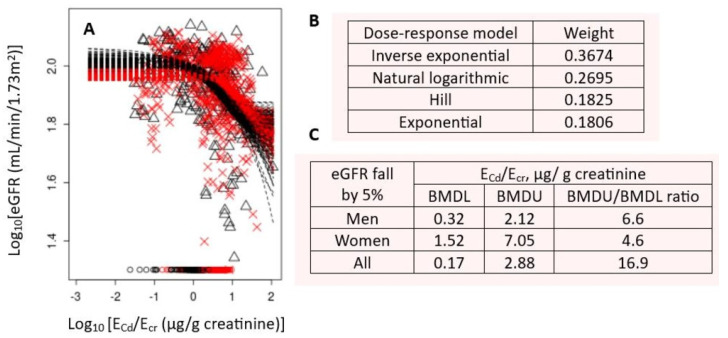
Cd excretion rate associated with a 5% reduction in eGFR. Bootstrap dose–response model averaging with 200 repeats for eGFR/E_Cd_/E_cr_ dataset (**A**), dose–response models and model weights (**B**) and BMDL/BMDU values for Cd excretion rates (**C**). × and △ represent male and female participants, respectively. Data were from 799 subjects (300 men and 499 women), aged 19–87 years (mean 49.2 years) [42].

Cd excretion rates among cohort participants ranged between 0.03 and 106 µg/g of creatinine (geometric mean 2.15 µg/g of creatinine). Age and BMI distributions conformed to a normal distribution [42]. The dose–response curves and benchmark Cd excretion rates using β_2_M and eGFR as endpoint measures are provided in Figure 4 and Figure 5, respectively.

The Cd excretion benchmark could not be reliably determined, as a 5% increase in β_2_M excretion was an endpoint, evident in the BMDU/BMDL ratio > 100 (Figure 4). In comparison, the benchmark Cd excretion rate of 0.17 µg/g of creatinine was obtained when a 5% reduction in eGFR was used as the endpoint (Figure 5). The BMDU/BMDL ratio of the benchmark Cd excretion was 16.9, meaning a high degree of certainty in the estimates. The use of the eGFR endpoint is recommended for future derivation of health-protective exposure limits for C because eGFR decline at a high rate signifies kidney disease.

#### 3.3.2. BMDL Values of Cd Excretion Derived from Human Population Data

Table 3 provides BMDL values of Cd excretion rates for different nephrotoxicity endpoints, using data from the general Chinese, Japanese and Thai populations.

*Conventional BMD*: Wang et al. employed a conventional BMD method to identify the BMDL values of Cd excretion at 5% and 10% prevalences of abnormal excretion of RBP, NAG and β_2_M [102]. The data used were from 934 (469 men, 465 women), aged 10–71 years, who were residents of Jiangshan City, Zhejiang, China [102]. For men, BMDL_5_ (BMDL_10_) values of Cd excretion were 0.89 (1.59), 0.62 (1.30), and 0.49 (1.04) μg/g of creatinine for the RBP, β_2_M, and NAG, respectively. Corresponding benchmark Cd excretion rates in women were 0.76 (1.53), 0.64 (1.34), and 0.65 (1.37) μg/g of creatinine.

Suwazono et al. identified Cd excretion rates of 0.6–1.2 and 0.6–2.3 µg g of creatinine as BMD values for abnormal β_2_M excretion in men and women, respectively [103]. They used data from 410 men and 418 women, aged 40–59 years, who lived in areas of Japan without Cd pollution [103]. The lower BMD estimates for abnormal β_2_M excretion in Japanese men and Japanese women were close to the BMDL_5_ values of Cd excretion in the Chinese study [102].

*Advanced BMD*: Satarug et al. used an advanced BMD method to determine Cd excretion benchmarks, with NAG excretion increased by 5% [104]. They used data from 734 Thai nationals (289 men and 445 women), 18–87 years of age (mean 48.1 years). Cd excretion benchmarks with 5% increase in NAG excretion in men and women were 0.060 and 0.069 µg/g of cr, respectively.

Using β_2_M excretion rates ≥ 300 μg/g creatinine as an endpoint, the BMDL_10_ values of Cd excretion were 0.469 and 0.733 µg/g of creatinine in men and women, respectively. A higher Cd excretion benchmark in women was a result of their universally smaller muscle mass than men; consequently, they have lower creatinine excretion rates than men. The Thai BMDL_10_ values of Cd excretion for β_2_M endpoint were 36–55% lower, compared to a Chinese study, reporting the BMDL_10_ values of 1.30 and 1.34 µg/g of cr in men and women, respectively [103]. This may be due to differences in age profiles of the target population and the cut-off value to define an abnormal β_2_M excretion, in addition to shortcomings of reliance on a single dose–response model in conventional BMD practice [106,107,108].

**Figure 6:**
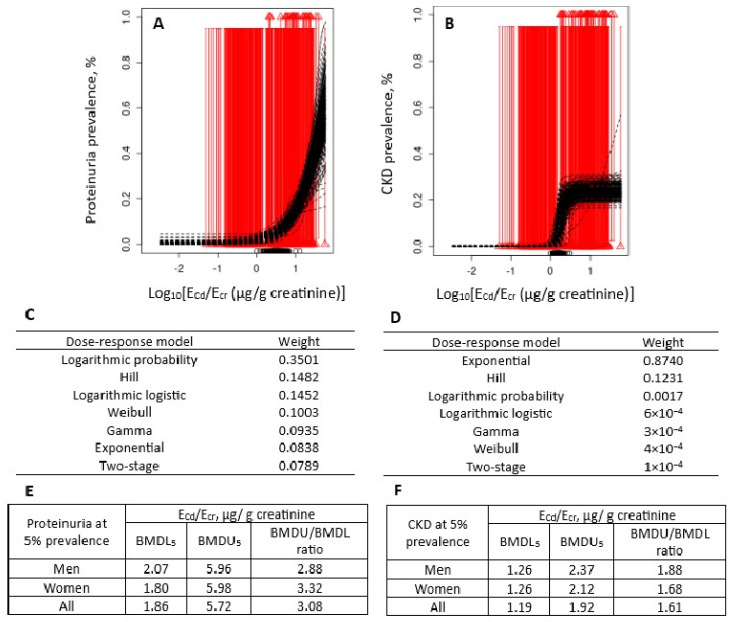
Cd excretion rates associated with 5% prevalences of proteinuria and CKD. Bootstrap dose–response model averaging with 200 repeats for proteinuria prevalence-E_Cd_/E_cr_ (**A**), CKD prevalence-E_Cd_/E_cr_ datasets (**B**), dose–response models and model weights (**C**,**D**), and BMDL/BMDU values for Cd excretion rates (**E**,**F**). Data were from 405 subjects (197 men and 208 women), aged 19–87 years (mean of 44.6 years) [105].

The advantage of using seven dose–response models to determine BMDL_5_ and BMDL_10_ values from dichotomous/disease prevalence data is illustrated in Figure 6.

Satarug et al. used the advanced BMD method to determine Cd excretion benchmarks at 5% prevalence of proteinuria and 5% prevalence of CKD [105]. The protein excretion rate of 100 mg/g cr. was a cut-off figure to define proteinuria, while the eGFR value ≤ 60 mL/min/1.73 m^2^ was a cut-off value to designate CKD development. The BMDL_5_ value of Cd excretion at 5% prevalence of proteinuria was 1.86 µg/g of creatinine, while the BMDL_5_ of Cd excretion at 5% prevalence of CKD was 1.19 µg/g of creatinine.

The BMDL value of Cd excretion at 5% CKD prevalence was 36% lower compared to the figure for 5% proteinuria prevalence. Apparently, a falling eGFR was a more sensitive indicator of Cd effects than proteinuria. Even a slight increase in Cd excretion (a body burden indicator of Cd) can induce a large drop in eGFR because the relationship between eGFR and Cd excretion was exponential (Figure 6D). This finding is consistent with a prospective cohort study from Switzerland that causally linked a fall of eGFR at a high rate (≥3 mL/min/1.73 m^2^/y) to Cd exposure [13].

Because proteinuria predicts continued eGFR deterioration [109,110,111], its clinical relevance is apparent. A health survey reported the prevalence of severe proteinuria (excretion of protein ≥ 200 mg/g cr) among residents of Mae Sot district to be as high as 24.1%, with 66.7% of them having Cd excretion rates ≥ 2 µg/g of cr [97].

The prevalence of proteinuria rose from 5% to 10% as Cd excretion rates rose from 1.86 to 4.47 µg/g of creatinine. In comparison, the prevalence of low eGFR rose from 5% to 10% as Cd excretion rates increased from 1.19 to 1.35 µg/g of creatinine; a 1.13-fold increase in Cd exposure resulted in 5% more people with CKD. In effect, the prevalence of CKD (low eGFR) was a more sensitive marker for Cd nephrotoxicity than proteinuria.

Collectively, benchmark Cd excretion limits suggested that Cd-induced nephron destruction that causes eGFR to fall has occurred in advance of a rise in protein excretion rates ≥ 100 mg/g of creatinine and a rise in β_2_M excretion rates above 300 µg/g of creatinine.

### 3.4. A Summary of Cd Toxicity Thresholds for the Kidney Target

Because the nephrotoxicity of Cd involves the same mechanism, the amount of Cd causing such toxicity can be expected to be similar across human populations. Accordingly, the BMDL (NOAEL equivalent) values of the Cd excretion rate estimated for various populations using the same endpoint, in theory, should be comparable.

For the nephrotoxicity endpoint, the lowest BMDL_5_ value of Cd excretion rate in a Chinese study using the NAG endpoint was 0.49 µg/g of cr (Table 3). In a Thai study using NAG excretion ≥ 4 U/g cr as a cut-off value, the lowest BMDL_10_ of Cd excretion was 0.469 µg/g of cr. The lowest Cd excretion benchmark identified in a Japanese study from the β_2_M endpoint was 0.6 µg/g of cr. These Cd excretion benchmarks are approximately 9–12% of the JECFA’s threshold.

Using a 5% decrease in the eGFR as an endpoint, the benchmark Cd excretion limit was 0.17 µg/g of cr. In comparison, the BMDL value of Cd excretion could not be reliably determined when 5% increase in β_2_M excretion was used as an endpoint. Intriguingly, the BMDL value of Cd excretion limit of 0.054 µg/g of cr. was derived for a 5% increment in total protein excretion. Similarly, the BMDL value of Cd excretion limit of 0.06 µg/g of cr. was derived for a 5% rise in NAG excretion [104], consistent with numerous studies suggesting NAG excretion to be sensitive to Cd [91]. However, unlike proteinuria, which predicts continued eGFR decline [109,110,111], the clinical utility of NAG has not been demonstrable. In a mediation analysis, NAG was minimally related to Cd-induced destruction of nephrons, which caused eGFR to fall [112]. This could be due to the fact that kidneys have the capacity to regenerate and repair the tubular cells following a mild-to-moderate injury induced by a low-dose Cd [113,114].

### 3.5. Established Dose–Response Relationships

The risk of CKD (eGFR < 60 mL/min/1.73 m^2^) has been related to dietary Cd exposure levels in a large Chinese population study (*n* = 8429) [115]. Compared with the dietary Cd exposure level of 16.7 μg/day, CKD risk rose 1.73-fold, 2.93-fold, and 4.05-fold as Cd intake levels rose to 23.2, 29.6, and 36.9 μg/day, respectively [115].

The risk of CKD (eGFR < 60 mL/min/1.73 m^2^) has been related to Cd excretion in a Thai population study, including 493 men and 696 women, with a mean age of 43.2 years and a mean Cd excretion rate of 0.64 µg/g of creatinine [101]. CKD risk rose 6.2- and 10.6-fold as urinary Cd excretion rate increased from 0.37 to 0.38–2.49 and ≥2.5 µg/g of creatinine, respectively [101]. These results were obtained after the potential confounders—age, BMI, gender and hypertension—were adjusted [101].

The above dose–response investigations have indicated an increment in CKD risk as a function of dietary Cd intake levels and Cd excretion rates [101,115]. Supporting these observations is the Swiss prospective cohort study, where a rapid fall of eGFR per year has been found to be caused by Cd exposure, adjusting for confounding factors [13]. These data provide a firm basis to derive exposure guidelines based on the eGFR endpoint. The factors influencing internal doses (blood Cd concentrations), and thus the toxic manifestation of Cd [116,117,118], could be integrated as safety margins.

## 4. Mechanism-Based Strategies to Mitigate Cd Toxicity

In this section, the current view around how Cd from foods reaches the kidney proximal tubular cells and mitochondrial target is highlighted, while accentuating the potential role for albumin in the delivery of Cd to kidney tubules. A summary is presented of the results from recent investigations advancing our knowledge on how Cd induces tubular cell death in chronic low-dose exposure conditions in humans, together with the strategies that can be developed to mitigate its toxic manifestations.

### 4.1. Why Does Most Acquired Cadmium Accumulate in the Proximal Tubules?

As depicted in Figure 2, Cd is absorbed by enterocytes via several transport proteins for Fe, Zn, Ca, and Cu. Similarly, Cd enters osteoblasts via multiple metal transport proteins for essential metals and voltage-gated Ca^2+^ channels [119]. In distinction from enterocytes and osteoblasts, the kidney tubular cells have the capacity to reabsorb proteins via receptor-mediated endocytosis (RME) [120,121,122], in addition to those for reuptake of metals, glucose, amino acids and all other nutrients.

Filtered Cd is primarily complexed with glutathione (GSH), inorganic anions, such as sulfate, phosphate and amino acids, particularly cysteine, and cystine. Being the principal segment of the nephron, where transcellular reabsorption of these filtered Cd occurs, the Cd load in the PT is highest [90]. The internalization of proteins other than Cd-MT complexes, notably albumin, can be an additional entry route for Cd [90,123,124].

Using mice exposed to Cd in drinking water for 1, 2, or 4 months, Fujishiro et al. observed a preferential accumulation of Cd in the kidney cortex, while noting MT was more abundant in the proximal tubules than in the distal tubules [125], similar to a study using human kidney sections [126]. Given that MT expression is induced by Cd, the high abundance of MT in proximal tubules compared to the distal tubules can thus be expected if PTCs absorbed most Cd.

### 4.2. Cd-Induced Albuminuria: Glomerular or Tubular Cause

With a large molecular weight (66 kDa) and negative charges, albumin is not filtered by glomeruli [127,128]. In normal health, 1–10 g of albumin (40–50 g of plasma protein) may reach the tubular lumen each day by means of transcytosis through endothelial cells and podocyte foot processes [129,130]. A majority of albumin is reabsorbed by fluid-phase endocytosis and returned to blood circulation by transcytosis, while a small fraction of albumin is reabsorbed through RME [127,128].

*Glomerular Membrane Permeability*: A four-fold increase in the glomerular filtration of albumin was noted in female Sprague-Dawley rats, exposed to Cd in drinking water for up to 18 months [131,132]. This glomerular membrane permeability effect of Cd appeared to occur in advance of GFR and tubular effects, evident from the excretion of enzymes, NAG, alanine aminopeptidase and lactate dehydrogenase [131]. Apparently, the glomerular membrane was particularly sensitive to Cd toxicity, resulting in increased membrane permeability to albumin. The permeability of human renal glomerular endothelial cells in monolayers was increased by Cd at 1 µM concentration [133], most likely due to the redistribution of the adherens junction proteins, vascular endothelial-cadherin and β-catenin [134].

**Figure 7:**
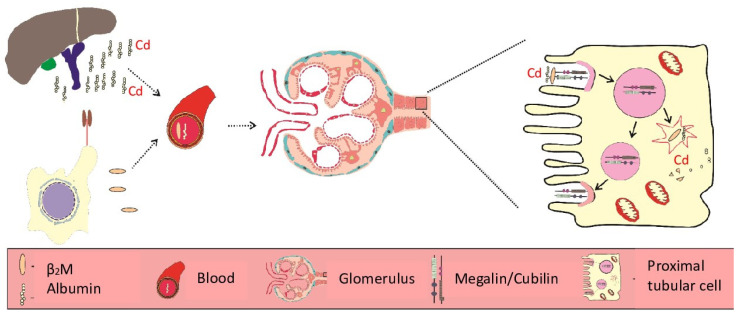
Reabsorption of β_2_M and albumin by the proximal tubular cell. Filtered β_2_M is reabsorbed totally via REM, followed by lysosomal degradation [91]. Only a small fraction of albumin is reabsorbed through RME, degraded in lysosome from which Cd is released [137,138], and subsequently bound to preexisting or nascent MT [90].

*Megalin/Cubilin RME*: In a study using data from 519 Thai subjects with moderate-to-high Cd exposure, the calculated fractional excretions were 0.18% for albumin and 21% for β_2_M. These calculations assumed the glomerular sieving coefficients to be 0.01 for albumin and 1 for β_2_M [135]. Because RME of β_2_M requires megalin but not cubilin, RME of albumin requires both proteins, it can be inferred that Cd had disrupted megalin-mediated endocytosis of both proteins by a single “shared” mechanism. Cd reduced expression levels of CUBN (cubilin) and LRP2 (megalin) in the kidney tubular cells was observed in a study using Sprague Dawley male rats given Cd in drinking water at 0, 50, or 75 mg/L CdCl_2_ for 1 and 6 months [136]. Filtered albumin not subjected to transcytosis in the S1 segment of the proximal tubule is processed identically to β_2_M (Figure 7).

The Cd released from lysosomal degradation of albumin is sequestered in cytosolic MT once it exits endo-/lysosomes via DMT1 [137,138]. The binding of Cd to MT in this manner is viewed as a detoxification mechanism. Notably, however, as the influx of Cd into PTCs continues, the metal binding sites of MT become saturated [17], leaving the metal in free ionic form (Cd^2+^) that can reach various cell organelles, especially the mitochondrion, which is characteristically abundant in PTCs [17,139]. These findings are consistent with the fact that excreted Cd is primarily in MT-bound form, a detoxified storage form of the metal [140,141], and excreted Cd reflects ongoing nephrotoxicity.

Because of the large number of mitochondria, the homeostasis and survival of PTCs rely heavily on autophagy [142,143,144]. Acute kidney injury in Cd-intoxicated rats was due to the inhibition of autophagy and interference with the function of lysosomes by Cd [145], while kidney fibrosis was due to impaired tubular protein endocytosis [109,110,111].

### 4.3. Albuminuria/Proteinuria and CKD

Albuminuria, defined as an albumin-to-creatinine ratio (ACR) of 20 mg/g cr in men and 30 mg/g cr in women, like eGFR, is a diagnostic criterion for CKD. However, its utility in CKD diagnosis is based mostly on circumstantial evidence; excretion of albumin at a rate of 7 mg/g of creatinine can predict CKD development within 10 years [146].

The effects of Cd on reabsorption of albumin and β_2_M were evident from analysis of data from 519 Thai subjects who were exposed to moderate-to-high levels of Cd [135] (Table 4).

Table 4 provides representative rates of filtration, excretion, catabolism and transcytosis in normal and Cd-intoxicated PTCs. Through megalin/cubilin RME, PTCs internalized a higher amount of albumin (3 g) than β_2_M (0.3 g) per day.

The role of oxidized albumin in causing kidney tubular cell death through ferroptosis has been demonstrated [147]. Prior to this observation, using a cell culture model of PTC, Fels et al. have demonstrated the cytotoxicity of Cd unambiguously complexed with albumin [123]. These findings underscore the significance of megalin/cubilin RME that mediates the internalization of Cd-albumin complexes/oxidized albumin by PTCs (Figure 7) [90,123,147], thereby providing additional evidence that connects impaired tubular protein reabsorption and/or impaired lysosomal protein degradation to albuminuria/proteinuria and kidney fibrosis [148].

The development of kidney fibrosis by a low-dose Cd exposure has been demonstrated in experimental studies [149,150]. Evidence of Cd-induced kidney fibrosis was noted in a synchrotron imaging of metals in human kidney tissue samples [151]. In a histopathological study, using kidney biopsies from kidney transplant donors, a correlation was observed between tubular cell atrophy and Cd accumulation levels [152].

Notably, however, studies deriving BMDL values using disease markers such as albuminuria/proteinuria are limited [107,153,154]. Most reported BMDL figures of Cd excretion were based on nephrotoxicity biomarkers (Section 2), which were not indicative of Cd early effects, nor were they clinically relevant.

### 4.4. CKD and Its Progression Toward End Stage

CKD is a major public health problem worldwide because it causes high morbidity and mortality, especially from cardiovascular disease [155,156], and kidney failure, signified by a fall of eGFR to 15 mL/min/1.73 m^2^ or below. Now, this ailment is ranked the seventh leading cause of global death, projected to rise further along with obesity, diabetes, hypertension, and non-alcoholic fatty liver disease. It will rise to the fifth leading cause of death in 2040 [157,158]. The cost associated with treatment involving dialysis and a kidney transplant is escalating.

Indisputably, exposure to Cd pollution has contributed to CKD development in a significant proportion of the adult population worldwide [11,12], while the Bradford–Hill Criteria strengthen a causal relationship between CKD and Cd exposure [3].

Concerningly, Cd-induced loss of eGFR is irreversible, and there is no effective chelation therapy to eliminate the kidney Cd load. Hence, developing strategies to prevent CKD development and to mitigate its nephrotoxicity is of great public health significance.

### 4.5. Cd and Cellular Stress Responses

Hijacking transport proteins for essential metals, Cd can enter any cell and reach its organelles and subcellular structures [139]. Thus, Cd can impact the function of nearly all cell types in the body [1,2], including erythrocytes [159] and leucocytes [160,161,162]. Through MT and transport proteins for Ca and Fe, i.e., the metal coupling unit (MCU) and DMT1, Cd reaches the inner membrane of mitochondria [163]; it reduces the synthesis of adenosine triphosphate (ATP), suppresses the electron transport chain [164], and promotes the formation of reactive oxygen species (ROS) [139,165,166]. The most frequently identified sign of Cd toxicity is related to oxidative stress damage to lipids and proteins, such as oxidized low-density lipoprotein and oxidized albumin, discussed in Section 4.3.

**Figure 8:**
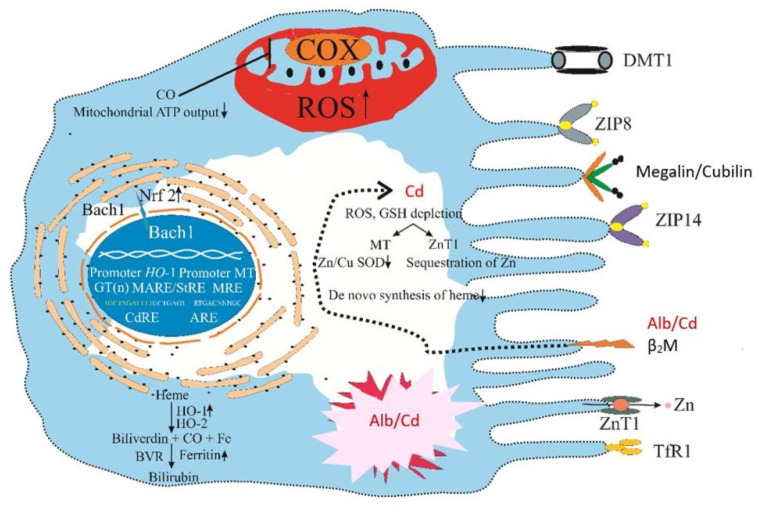
Uptake and accumulation of Cd by the kidney proximal tubular cell. Many specialized transport proteins are expressed by the proximal tubular cells (PTCs), which enable them to retrieve all nutrients, i.e., glucose, amino acids, metals and filtered proteins from filtrate. The receptor-mediated endocytosis (RME) involving megalin/cubilin is responsible for protein internalization, β_2_M, and albumin [122,123]. Reabsorption of Cd-MT complexes occurs in the distal and collecting tubules, involving SLC22A17/lipocalin-2 receptor [124]. There is little evidence that Cd-MT is reabsorbed by megalin/cubilin RME [90]. Upward (↑) and downward (↓) arrows indicate rising/falling expression levels and catalytic activities.

In addition to mitochondrial source, ROS can be produced, normally in the peroxisomes, and the endoplasmic reticulum [167]. To maintain cellular redox states and cell function, as well as to protect against potential damage from excessive ROS formation, many mechanisms have evolved. Herein, antioxidant defenses involving the upregulation of the *MT* gene and the *heme oxygenase-1 (HO-1)* genes by cells in response to Cd are highlighted (Figure 8).

#### 4.5.1. Cd-Induced Upregulation of the MT Gene

Upregulation of the MT gene by PTCs in response to Cd is a detoxification mechanism because sequestering of Cd in MT (Cd-MT complexes) prevents acute toxicity due to “free” Cd^2+^ ion which is the chemically reactive form of the metal. Each MT molecule can carry up to 7 atoms of Cd^2+^, 7 atoms of Zn^2+^ or 12 atoms of Cu^2+^, and the metal–MT complexes are denoted as Cd_7_MT, Zn_7_MT, and Cu_12_MT [168]. Complexes of mixed metals, such as Cd_3_Cu_3_ZnMT, Cd_4_CuZn_2_MT, and Cd_6_CuMT, are formed in vivo [168].

Approximately 16 MT isoforms have been identified in four major families, MT-1–MT-4 [169]. MT-1 and MT-2 are ubiquitously expressed by most cells, including leucocytes and kidney tubular cells [122,170,171,172]. MT-3 has a particularly high binding affinity for Cu, and high levels of MT-3 isoform are found in kidneys and neurons [138,172,173]. Because free Cu ion is redox active, Cu-MT-3 complexes may account for the nephrotoxicity and neurotoxicity of Cd [173].

A study from Taiwan (*n* = 2447, mean age 55.1 years) [174] noted that subjects with proteinuria had a mean urinary Cd concentration 27.3% higher than those without and that the risk of proteinuria was increased 2.67-fold and 1.94-fold in those with elevated urinary Cd and Cu levels, respectively. Proteinuria was found in Taiwanese subjects with low exposure levels, reflected by a mean urinary Cd in subjects of 1.1 μg/L. This urinary Cd level was in line with the proteinuria-based BMDL value of Cd excretion.

A direct correlation between 24 h proteinuria and urinary copper levels was observed, together with an inverse correlation of eGFR and serum Cu in a study that included 313 kidney disease patients and 19 healthy controls [175]. These data explain a connection between urinary Cu and proteinuria observed in the Taiwanese study [174].

Because absorbed Cd reaches the liver first, Cd-MT complexes formed in the liver presumably contain oral Cd, while those synthesized in the lungs contain inhaled Cd. Liver and lungs serve as endogenous reservoirs from which Cd-MT complexes are released as cells die. Cd–MT complexes are redistributed to the kidneys, which are equipped with protein internalization.

There is little evidence that megalin/cubilin RME in the proximal tubule reabsorbs the complex of Cd-MT at concentrations expected in the glomerular ultrafiltrate [90]. However, it could be retrieved in the distal tubule and collecting duct by SLC22A17/lipocalin-2 REM [122,124]; consequently, filtered Cd-MT complexes are all absorbed and retained within those regions of nephrons.

Sequestering Cd via MT prevents acute cytotoxicity; however, it may increase the risk of long-term toxicity because Cd^2+^ ions can be released under certain conditions, leading to increased synthesis of nitric oxide (NO) that liberates the Cd and Cu previously bound to MT [176,177,178]. Moreover, upregulation of MT in response to Cd can impact cellular redox state, vital to maintaining normal protein structures, intermediary metabolism, and cell function [179,180,181]. For example, increased sequestration of Zn and Cu in MT could lead to a reduction in the activity of the antioxidant enzyme superoxide dismutase 1 (SOD1) that requires Zn and Cu as cofactors [182].

#### 4.5.2. Cd-Induced Upregulation of the HO-1 Gene

In response to any form of stress, including hyperglycemia, the HO-1 gene is upregulated [183]. Unexpectedly, however, HO-1 gene activation by Cd appeared to be different from physiological HO-1 regulators, i.e., prostaglandin D_2_ (PGD_2_) [184]; as such, induction of HO-1 expression by Cd did not result in a concomitant increase in bilirubin synthesis [185]. The reason for this phenomenon remains unclear.

Satarug et al. have shown that PGD_2_ activated the HO-1 gene in a cell culture model of human retinal pigment epithelial cells through the D-prostanoid_2_ (DP_2_) receptor [180]. In contrast, Cd activated the HO-1 gene via two enhancers: the Cd response element (*CdRE*, TGCTAGATTTT) and Maf recognition antioxidant response element (*MARE*, GCTGAGTRTGACNNNGC), also known as *ARE* or the stress response element (*StRE*) [186]. Moreover, Cd induced nuclear export of the repressor Bach1 and suppressed the lysosomal degradation of Nrf_2_ [187], resulting in the transactivation of the HO-1 gene by the Nrf_2_/small Maf complex [188].

The potency of Cd to activate the HO-1 gene in various human cell lines has been determined using the *ARE* reporter gene assay [189] (Table 5).

As shown in Table 5, through a single enhancer *ARE*, the expression of HO-1 in human kidney and liver cell lines is increased by Cd as little as 1 µM. Cd and arsenic (As) both activate HO-1 expression in all five human cell lines tested. In the human breast cancer cell line, MCF7, the HO-1 induction potency of Cd was comparable to that of As. In all other cell lines, the HO-1 induction potency of Cd was higher than that of As. Notably, the induction of HO-1 by As, like Cd, did not lead to an increase in bilirubin concentrations in Cd- and As-exposed cells [185]. The signaling pathways involved in cellular responses to various forms of stress warrant further investigation.

Through *CdRE* and *ARE*, HO-1 expression and catalytic activity are increased immensely, leading to a degradation of heme from which Fe is released, while carbon monoxide (CO) is generated. Nonetheless, there is a little change in bilirubin concentrations in cells treated with Cd [185]. Bilirubin, by virtue of its lipophilic properties, protects lipids from oxidation more effectively than the water-soluble antioxidants, such as GSH and vitamin C [190]. A massive induction of HO-1 gene expression by Cd can lead to loss of PTCs through ferroptosis [191,192,193,194].

Ferroptosis is an iron-dependent regulated cell death, a consequence of overt lipid peroxidation [195]. Like tubular cells of the kidneys, Cd-induced cell death via ferroptosis occurred in cells of various tissues and organs [196] such as bones [197], ovaries [198] and testes [199,200]. These observations are consistent with the fact that an elevation in HO-1 expression is a universal cellular response to a broad range of stressors, including physical (shear stress), metabolic, xenobiotic, and biological stressors [183]. In the absence of a concomitant increase in bilirubin formation, Cd-induced HO-1 expression can result in lipid peroxidation and ferroptosis, given that HO-1 is localized in the endoplasmic reticulum.

### 4.6. Can Exogenous HO-1 Inducers Mitigate Cd-Induced Oxidative Stress?

As discussed above, Cd appears to cause oxidative stress through lowering levels of endogenous antioxidants, especially bilirubin. This raises the possibility of replenishing such bilirubin insufficiency by exogenous HO-1 inducers. A wide range of antioxidants from plant foods, such as curcumin, quercetin, tert-butylhydroquinone, and caffeic acid phenethyl ester, are known to be HO-1 inducers, as are catechin (in green tea), α-lipoic acid (in broccoli, spinach), resveratrol (in red wine, grapes), carnosol, sulforaphane (cruciferous vegetable), coffee diterpenes cafestol, and kahweol [201]. The beneficial effects of the consumption of these plant antioxidants could thus be mediated in part through the induction of HO-1 expression.

A diet high in anti-oxidative and anti-inflammatory nutrients was associated with increased serum bilirubin levels and reduced oxidative stress and systemic inflammation associated with Cd exposure [202]. Furthermore, the consumption of plant-based diets may provide a viable option to prevent and manage CKD, which continues to rise worldwide [203,204,205]. The investigation into the potency of plant-derived compounds to activate the HO-1 gene using the ARE reporter gene assay or similar constructs should be encouraged [206]. Interestingly, hypobilirubinemia (<10 μM) is an independent risk factor for cardiovascular-kidney-metabolic (CKM) syndrome [207,208]. In a recent preclinical study, suppression of gut bacterial conversion of bilirubin to urobilin could be another strategy to raise plasma bilirubin concentrations [209].

## 5. Conclusions

Current dietary Cd exposure guidelines vary fourfold, ranging from 0.21 to 0.83 μg/kg b.w./d with a tenfold difference in threshold levels of Cd excretion, varying between 0.5 and 5.24 µg/g of creatinine. These exposure guidelines derived by JECFA, EFSA, US FDA and ATSDR, respectively, described as TDI, RfD, TRV and MRL, were all based on the premise that there is a critical exposure level below which adverse effects of Cd on bone/and or kidney target are discernible. Notably, however, the existing NOAEL/BMDL values of Cd excretion derived for the kidney target were based on nephrotoxicity indicators, not kidney disease, which is diagnosed when eGFR falls to a third of normal value, or ACR rises to 20 mg/g cr in men and 30 mg/g cr in women for at least 3 months.

The benchmark Cd excretion limit could not be reliably determined when a 5% increment in β_2_M excretion was used as an endpoint. The benchmark Cd excretion limit at 5% increases in protein excretion is as little as 0.054 µg/g of creatinine [105]. Thus, there is no basis for using β_2_M excretion in computing the health risk due to dietary Cd exposure.

The benchmark Cd excretion limit derived at 5% drop in the eGFR is 0.17 µg/g of creatinine [42]. The certainty of this BMDL estimate is high, indicated by a BMDU/BMDL ratio of 16.9. A rapid fall of eGFR as a consequence of Cd exposure noted in the Swiss prospective study [13] supports the use of an eGFR-based threshold. Also, the BMDL_5_ (BMDL_10_) values of 0.198 (0.365) μg/g creatinine, derived from the prevalence of diabetes in 4530 US adults [210], align with the eGFR-based threshold. Using Chinese population data, the eGFR-based reference value of dietary Cd exposure is 0.149 µg/kg b.w./d for adults and 0.018 µg/kg b.w./d for children [211].

## Figures and Tables

**Table 1:** Comparison of international Cd exposure guidelines and derived thresholds.

Toxicity Target/Endpoint	Exposure Limit/Threshold	Reference
Kidneys/β_2_M excretion ≥ 300 µg/g of creatinine	TDI of 0.83 μg/kg b.w./d 5.24 μg/g creatinine	JECFA, 2010 [61,62]
Kidneys/β_2_M excretion ≥ 300 µg/g of creatinine	RfD of 0.36 μg/kg b.w./d 1 μg/g creatinine	EFSA, 2011 [63]
Kidneys and bones/ β_2_M excretion and bone mineral density	TRV of 0.21−0.36 μg/kg b.w./d 0.5 μg/g creatinine	US FDA, 2023 [64]
Bones/bone mineral density	MRL of 0.5 µg/kg b.w./d for an intermediate exposure duration (15−365 d)	ATSDR, 2017 [43]
Lungs/alveolar histiocytic infiltration and focal inflammation in alveolar septa	MRL of 0.03 μg CdO/m^3^ for an acute exposure duration (1−14 d)	ATSDR, 2017 [43]

β_2_M, β_2_-microglobulin; TDI, tolerable daily intake; RfD, reference dose; TRV, toxicological reference value; MRL, minimal risk level; CdO, cadmium oxide.

**Table 2:** Estimated cadmium exposure levels through the diet.

Foods	Cd, mg/kg		Intake, g/d	Exposure, µg/d	
	Maximum	Typical		High	Average
Vegetables; potatoes, included	0.1	0.05	250	25	12.5
Cereals, pulses, legumes; rice, wheat grain included	0.2	0.05	200	40	10
Fruit	0.05	0.01	150	7.5	1.5
Oilseeds and cocoa beans	1.0	0.5	1	1	0.5
Meat; cattle, poultry, pig, sheep	0.1	0.02	150	15	3.0
Liver; cattle, poultry, pig, sheep	0.5	0.1	5	2.5	0.5
Kidney; cattle, poultry, pig, sheep	2.0	0.5	1	2	0.5
Fish	0.05	0.02	30	1.5	0.6
Crustaceans, mollusks	2	0.25	3	6	0.75
TOTAL	−	−	−	93.5	30

Food types and consumption rates were based on a typical Australian diet [88]. The mean dietary Cd exposure among Australian children, aged 8 years, was 60% of JEFCA’s TDI [88].

**Table 3:** Benchmark cadmium excretion rates for nephrotoxicity and CKD development.

Method/Endpoint	Cd excretion Benchmark	Country/ Reference
Conventional BMD RBP, β_2_M, and NAG	For men, BMDL_5_ (BMDL_10_) values for Cd excretion rates with abnormal excretion of RBP, β_2_M, and NAG were 0.89 (1.59), 0.62 (1.30), and 0.49 (1.04) μg/g cr., respectively Corresponding BMDL_5_ (BMDL_10_) values for Cd excretion rates in women were 0.76 (1.53), 0.64 (1.34), and 0.65 (1.37) μg/g cr.	China, Wang et al. [102]
Conventional BMD β_2_M	Respective BMD values of Cd excretion rates with abnormal β_2_M excretion in men and women were 0.6–1.2 and 0.6–2.3 µg g cr. [102].	Japan, Suwazono et al. [103]
Advanced BMD β_2_M, NAG	Cd excretion benchmarks at 5% increase in NAG excretion in men and women were 0.060 and 0.069 µg/g of cr., respectively. BMDL_10_ value of Cd excretion rate at 10% prevalence of β_2_M excretion rates ≥ 300 μg/g cr. were 0.469 and 0.733 µg/g of cr. in men and women, respectively.	Thailand, Satarug et al. [104]
Advanced BMD Total protein, eGFR	Cd excretion benchmark at 5% (10%) increase in protein excretion was 0.054 (0.114) µg/g of cr. BMDL_5_ (BMDL_10_) value of Cd excretion at 5% (10%) prevalence of CKD was 1.19 (1.35) µg/g of cr. BMDL_5_ (BMDL_10_) value of Cd excretion at 5% prevalence of proteinuria was 1.86 (4.47) µg/g of cr.	Thailand, Satarug et al. [105]

RBP, β_2_M, β_2_-microglobulin; NAG, N-acetyl-β-D-glucosaminidase; cr, creatinine; eGFR, estimated glomerular filtration rate. CKD was defined as eGFR values ≤ 60 mL/min/1.73 m^2^. Proteinuria was defined as excretion of protein rates ≥ 100 mg/g cr.

**Table 4:** Estimated amounts of albumin and β_2_M reabsorption through RME.

PTC Status	Protein	Filtration Rate	Excretion Rate	Catabolic Rate	Transcytosis Rate
Normal	Albumin	60 g/d	20 mg/d	2.980 g/d	57 g/d
	β_2_M	300 mg/d	100 μg/d	299.9 mg/d	0
Cd-intoxicated	Albumin	60 g/d	0.5 g/d	2.950 g/d	57 g/d
	β_2_M	300 mg/d	1000 μg/d	299 mg/d	0

Assumptions: plasma albumin is 40 g/L; plasma β_2_M is 2.0 mg/L; GFR is 150 L/d; the glomerular sieving coefficient for albumin (GSC_alb_) is 0.01; and GSC_β2M_ is 1 [135].

**Table 5:** The potency of Cd to induce the HO-1 gene upregulation assessed by *ARE* reporter assay.

Metal	Kidney (HEK293T)	Liver (HepG2)	Breast (MCF7)	Brain (A172)	Lung (A549)
Cd	0.907	0.954	11	6.03	54.7
As	1.88	16.5	9.05	15.9	207
Hg	2.82	19.5	6.35	6.18	NR
Pb	#	426	#	#	NR
Ag	11.8	2.52	4.73	5.54	NR
Au	76.1	169.7	40	146	NR
Zn	84.8	249	256	100	NR
Cu	281	455	295	136	392
Co	484	185	532	NR	NR
Fe	#	#	NR	239	NR

#, an unreliable result; NR, no response. Numbers are µM concentrations of individual metals that induce an increase in the HO-1 gene expression via the *ARE* [189].

## Data Availability

No new data were created or analyzed in this study. Data sharing is not applicable to this article.
